# Forecasting the Future Risk of Barmah Forest Virus Disease under Climate Change Scenarios in Queensland, Australia

**DOI:** 10.1371/journal.pone.0062843

**Published:** 2013-05-15

**Authors:** Suchithra Naish, Kerrie Mengersen, Wenbiao Hu, Shilu Tong

**Affiliations:** 1 School of Public Health, Queensland University of Technology, Queensland, Australia; 2 Mathematical Sciences, Queensland University of Technology, Queensland, Australia; Northeastern University, United States of America

## Abstract

**Background:**

Mosquito-borne diseases are climate sensitive and there has been increasing concern over the impact of climate change on future disease risk. This paper projected the potential future risk of Barmah Forest virus (BFV) disease under climate change scenarios in Queensland, Australia.

**Methods/Principal Findings:**

We obtained data on notified BFV cases, climate (maximum and minimum temperature and rainfall), socio-economic and tidal conditions for current period 2000–2008 for coastal regions in Queensland. Grid-data on future climate projections for 2025, 2050 and 2100 were also obtained. Logistic regression models were built to forecast the otential risk of BFV disease distribution under existing climatic, socio-economic and tidal conditions. The model was applied to estimate the potential geographic distribution of BFV outbreaks under climate change scenarios.

The predictive model had good model accuracy, sensitivity and specificity. Maps on potential risk of future BFV disease indicated that disease would vary significantly across coastal regions in Queensland by 2100 due to marked differences in future rainfall and temperature projections.

**Conclusions/Significance:**

We conclude that the results of this study demonstrate that the future risk of BFV disease would vary across coastal regions in Queensland. These results may be helpful for public health decision making towards developing effective risk management strategies for BFV disease control and prevention programs in Queensland.

## Introduction

As global climate change becomes unequivocal, there is increasing scientific interest in the assessment of its potential effects on human health, particularly, on the spread of mosquito-borne diseases [1]. Mosquito-borne disease transmission depends on mosquito biology and population dynamics, which itself depends on climate for several purposes: mosquitoes require water to breed and warm temperature is important for larval development and adult feeding behaviour. Although a suitable climate (i.e., temperature, rainfall and humidity) is necessary for disease transmission, other factors are also associated with disease outbreaks, including virus, vector and susceptible host [2].

Barmah Forest virus is the second important mosquito-borne disease in Australia, with several hundreds of clinically confirmed cases each year (for example, 1,855 people in 2011) [3]. It is transmitted predominantly by *Aedes* and *Culex* species of mosquitoes [2]. No effective vaccine or treatment is yet available, so the prevention and management of disease has solely relied on vector control programs, for example, reduction of breeding sites, public health education and the use of insecticides [4]. These programs have succeeded in eradicating mosquitoes in some areas, but have proved difficult to maintain in a long-term [5].

Evidence suggests that mosquito-borne diseases may be predicted by climate-based statistical models [6]. Some statistical models were developed to project future transmission of mosquito-borne diseases under climate change scenarios [7]. However, these models did not adequately account for interactions between climate variables and non-climatic factors [8]. An empirical model of future risk of malaria transmission, which accounted for interactions between climate variables [9], predicted little changes in the global distribution of the risk population by the year 2050. However, a mathematical model of dengue risk transmission, which considered temperature, rainfall and vapour pressure as exposure variables, individually, and in combination, with or without statistical interactions terms, predicted 20–30% increase in changes in the distribution of the risk populations by the year 2085 [9]. In order to predict the potential future risk of BFV disease in response to predicted climate change, it is important to assess the association between climatic and socio-economic factors and BFV disease [10], [11], to facilitate the planning and implementation of BFV disease control and prevention programs.

The projected level of global warming for the year 2030 (0·6–1·5°C) using A1B emission scenario is anticipated to cause changes in the distribution and pattern of mosquito-borne diseases in Australia [12]. Whether these changes result in increased or decreased numbers of disease outbreaks will depend on several abiotic and biotic factors at local and regional levels. Working knowledge of where BFV disease outbreaks will potentially occur in the future under climate change scenarios is essential for BFV disease risk management. In this study, we assessed the current geographical distribution of BFV disease transmission across Queensland coastal regions. Then, we projected the potential changes in the risk of geographical distribution of BFV disease transmission for the years 2025, 2050 and 2100 in Queensland, Australia, using the medium level A1B climate change scenario.

## Methods

### Study area

The study was conducted in Queensland, Australia, covering an area of 1,727,200 km^2^ (22·5% of the country) with 7,400 km of continental coastline and 9,800 km including islands. The estimated population was 4,580,725 on 30 June, 2011 [13]. Queensland has frequently recorded the largest outbreaks of BFV disease compared to any other Australian State. For example, in 2010, the annual average incidence rate (20·1/100,000 population) of Queensland is three times higher than the national annual average incidence rate (6·6/100,000 population) [3]. The climate in Queensland varies markedly, ranging from hot arid temperate, through warm wet tropical coastal belt, to mild subtropical zone. With this diversity, it demonstrates different degrees of spatial variability, particularly with regard to rainfall [14].

### Ethics statement

The study was approved by the Data custodians, Human Research Ethics Committee under Chapter 6, Part 4, Section 280 of the Public Health Act 2005, Communicable Diseases Branch in the Queensland Health and following the ethical considerations of the Research Ethics Unit, Queensland University of Technology (approval number: 0900000388).

### Data collection

The data on notified BFV cases covering the period 2000–2008 were obtained from Queensland Health. Gridded (5 km×5 km) climate data with complete records of annual average maximum and minimum temperature and rainfall were provided by Australian Bureau of Meteorology [14] for the same period. Data on socio-economic indicator (i.e., SEIFA index) [13] and tides [15] were obtained from Australian Bureau of Statistics and Queensland Transport, respectively. The geographic regions used for this analysis are mesh blocks which cover Queensland without gap or overlap. The mesh block population data including number of dwellings and overall population were supplied by Australian Bureau of Statistics [16] and these were used in the computation of BFV disease incidence rates. Located in the north-eastern corner of Australia, the state is divided into 60,758 spatial mesh blocks, with most residential mesh blocks containing 30–60 dwellings. Data were entered into a geographical information system database format.

Future climate projection data were downloaded for the years 2025, 2050 and 2100. The data included average annual maximum temperature, average annual minimum temperature and total annual rainfall. We adopted Commonwealth Scientific and Industrial Research Organisation (CSIRO) [17] using CSIRO: Mk3·5 climate change pattern with Special Report on Emissions Scenario Marker Scenario A1B (medium CO_2_ emissions, peaking around 2030) in this study because Intergovernmental Panel on Climate Change (IPCC) forecast that Australia would come under mild climate emissions [18].

### Statistical analyses

#### Model building

Firstly, we examined the associations between BFV disease incidence and climatic, socio-economic and tidal variables. Multi-collinearity was checked before entering these variables into the model. Multivariable logistic regression models were constructed to predict the probability of BFV disease outbreak (presence = one or absence = zero) (mean+1SD) [19]. Spatially autocorrelated BFV incidence after accounting for spatial dependence and heterogeneity was logarithmic transformed prior to outbreak classification due to skewed distribution. We used climatic variables (maximum and minimum temperatures and rainfall), SEIFA index, low tide and high tide as predictors.

Akaike information criterion (AIC) was employed to determine the best-fit model [20]. The accuracy, sensitivity and specificity values were also estimated using SPSS [21]. Area under the curve (AUC) of the receiver operating characteristic (ROC) was used to determine discriminatory performance of the model predictions relative to observed outbreaks. An AUC value of 0·8 was used as good and an acceptable predictive performance [22]. The results were considered statistically significant at p<0·05.

#### Projections of future risk

As recommended by IPCC [18], the best-fit model results were applied to future climate change scenarios to generate projections of BFV disease risk in the years 2025, 2050 and 2100. The output model was transformed into probability risk maps yielding the geographic distribution of current and future BFV disease outbreaks for entire coastal regions (<100 km distance away from coastline) [23].

The output of the model resulted in a risk probability of BFV disease for each mesh block. A probability value close to zero indicates the location with low risk of BFV disease outbreaks whereas a probability value of one suggests the area with high risk of BFV disease outbreaks. These estimated risk probabilities of BFV disease were mapped for 2000–2008, and then, the baseline risk probabilities were applied to project the future risk for years 2025, 2050 and 2100 under climate change scenarios.

## Results

### Regression model

Table 1 shows summary of the model results for the current distribution of BFV disease (Table 1). The best-fit model included minimum temperature, rainfall, and an interaction term between these two variables, SEIFA index and low tide. Model had a high accuracy of 90·2%, sensitivity of 98·0% and specificity of 88·4%. The model also showed a high predictive performance with 0·98 AUC-ROC value (Table 1).

**Table 1:** Odds ratios of climate, socio-economic and tidal variables associated with BFV disease outbreaks in the entire coastal region in Queensland.

Variables	Odds ratio	95% CI	P-value
Minimum temperature (°C)	1·61	1.368–1.888	0·00
Rainfall (mm)	1·04	1.036–1.044	0·00
Low tide	0·01	0.000–0.001	0·00
SEIFA	1·01	1.005–1.007	0·00
Minimum temperature * Rainfall	0·99	0.997–0.998	0·00
Constant	0·00	-	0·00

Accuracy: 90·2%; Sensitivity: 98·0%; Specificity: 88·4%.

### Future regions at risk

**Figure 1:**
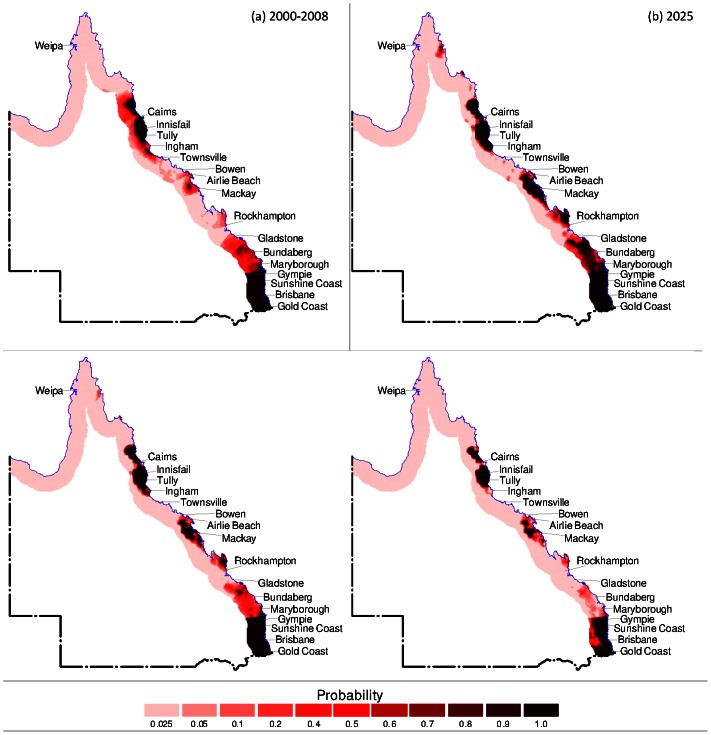
Forecast of BFV disease setting minimum temperature constant. (a) Geographical distribution of BFV disease under current climatic conditions for Queensland entire coastal regions, (b) forecast of potential probabilities of risk of BFV disease under climate change scenarios setting minimum temperature constant for 2025, (c) 2050 and (d) 2100.

**Figure 2:**
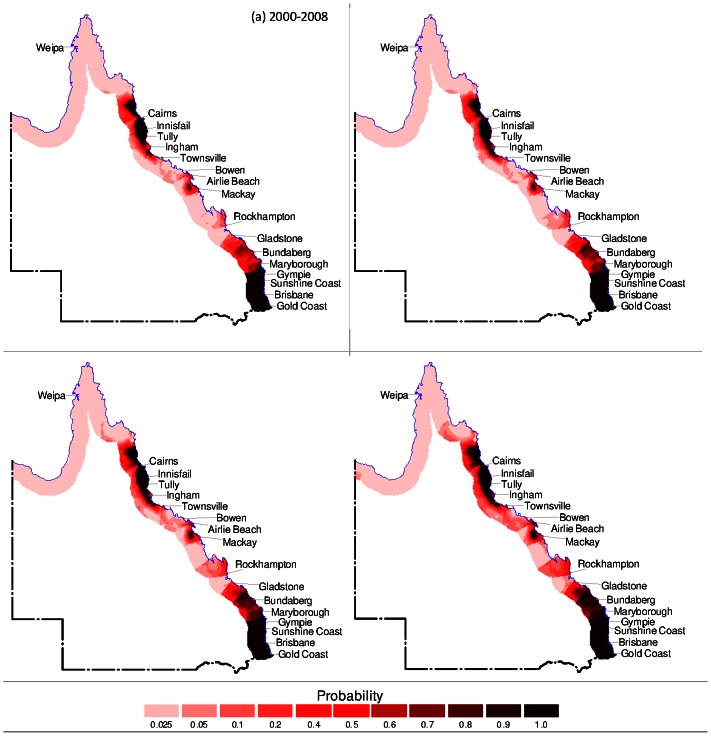
Forecast of BFV disease setting rainfall constant. (a) Geographical distribution of BFV disease under current climatic conditions for Queensland entire coastal regions, (b) forecast of potential probabilities of risk of BFV disease under climate change scenarios setting rainfall constant for 2025, (c) 2050 and (d) 2100.

**Figure 3:**
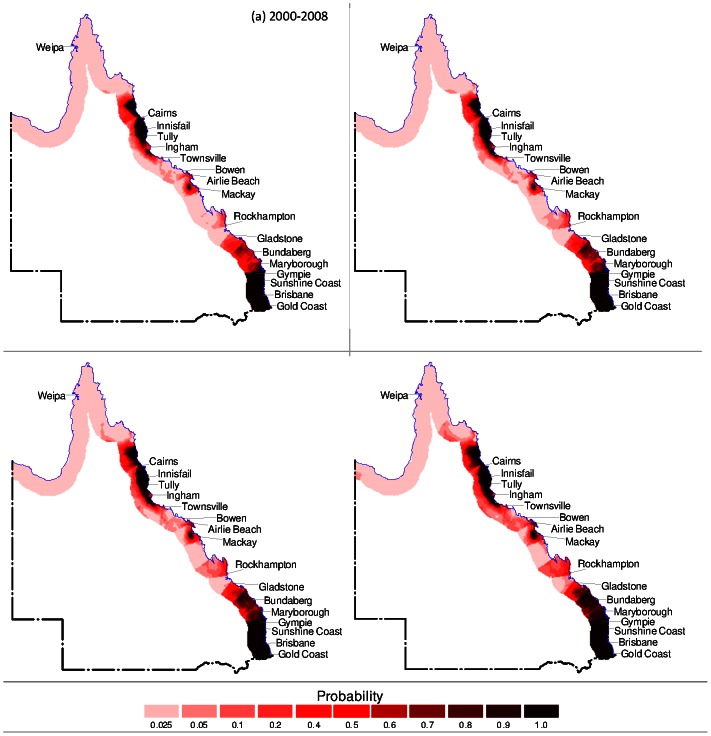
Forecast of BFV disease setting minimum temperature and rainfall varies. (a) Geographical distribution of BFV disease under current climatic conditions for Queensland entire coastal regions, (b) forecast of potential probabilities of risk of BFV disease under climate change scenarios for 2025, (c) 2050 and (d) 2100.

In order to visualise the future regions at risk, the results of the logistic regression model were mapped for existing climatic, socio-economic and tidal conditions. The model was then used to forecast BFV risk for years 2025, 2050 and 2100 under climate change scenarios. Only predicted rainfall and minimum temperature were used as the changing variables as socio-economic and tidal data were not available for these future years. The model was used firstly to forecast BFV risk assuming that minimum temperature remains constant based on existing conditions (Figure 1). Secondly, forecasting of BFV risk was conducted assuming that the rainfall remains constant based on existing conditions (Figure 2). Finally, Figure 3 presents the forecasting of BFV risk assuming that both rainfall and minimum temperature will change in future years compared to existing conditions.

Figure 1 (a) presents BFV risk under existing conditions. Figure 1 (b), (c) and (d) shows the forecast results for years 2025, 2050 and 2100 respectively due to varying rainfall but constant minimum temperature. Similarly, with constant rainfall and varying minimum temperature, Figure 2 (b), (c) and (d) shows the forecast results for years 2025, 2050 and 2100 respectively. Likewise, Figure 3 (b), (c) and (d) shows the forecast results for years 2025, 2050 and 2100 with both rainfall and minimum temperature varying.

In all forecast scenarios, the following can be observed. Under existing conditions there are areas of very high risk (P≅1·0) around Brisbane area (including Gold Coast and Sunshine Coast) and Cairns area (including Ingham and Cooktown, approximately 100 km north of Cairns). Compared to the entire coastal region these two areas demonstrate the relatively highest levels of risk. In year 2025 the high risk areas are the same as for existing conditions (Brisbane and Cairns) but also include emerging areas such as Mackay and Rockhampton and between Gladstone and Gympie. BFV risk remains very high (P≅1·0) around Brisbane, Cairns and Mackay and Rockhampton areas for years 2050 and 2100. However, in all other areas along coastal Queensland BFV risk progressively decreases from 2025 to 2100. The scenario where both rainfall and minimum temperature varies in future years (Figure 3 (b), (c) and (d)) produces the nearly similar patterns of BFV risk spread across coastal regions in Queensland compared to the other two assumptions, however BFV risk decreases more significantly. Some possible reasons for this are presented in the discussion, however it is also likely that the sensitivity of the logistic regression model is very high. Thus minor changes in both varying rainfall and minimum temperature produces a noticeably different risk level compared to only one variable varying.

## Discussion

This study projected future risk of BFV disease outbreaks under climate change scenarios in coastal regions, Queensland, Australia. To be useful for disease surveillance and control programs, a distribution geographic risk model should: (1) use predictors that are easily available and interpretable; (2) be accurate against independent data; and (3) generate outputs that can assist control decisions [24]. In this study, we developed a robust predictive model which included climatic, socioeconomic and tidal variables. Then, we projected the future risk of BFV disease based on the combination of the baseline model and climate change emission scenarios [12], [17].

We have produced an empirical model for projecting future BFV risks across Queensland coastal regions, highlighting the variability of various factors (including climatic, socio-economic and tidal) influencing BFV outbreaks. This is consistent with findings that mosquito-borne disease transmission is determined by multiple factors [1], [2]. Other factors such as wetlands may also influence BFV disease outbreaks, and hence we have included these variables and the model performances did not vary much. Consequently, the final model did not include wetland variables as these did not affect the accuracy of the model. So we used a parsimonious model. Logistic regression modelling is a useful tool for interpreting and applying surveillance data. It has a great potential to be used as a decision-support tool in mosquito-borne diseases. On the assumption that other factors affecting BFV outbreaks remain constant over time, we forecast that climate change would affect future risk of BFV disease across coastal Queensland. The finding that the baseline distribution of BFV disease is well predicted by temperature, rainfall and an interaction between these two variables is biologically plausible [1], [2]. Both temperature and rainfall are important for breeding and survival of mosquito populations. Previous research indicates that mosquitoes that transmit mosquito-borne disease are sensitive to temperature. Evidence also has accumulated to show that heavy rainfall and flooding can lead to increased mosquito breeding and outbreaks of mosquito-borne diseases in Australia. Our study corroborated previous studies [10], [11] and indicated that climate is one of the key predictors of BFV outbreaks.

Evidence suggests that the future mosquito-borne disease transmission will also depend on socio-economic and tidal factors [10], [11]. Hence, we included socio-economic indicator and tides in the development of baseline models for predicting BFV outbreaks. Our results indicate that SEIFA index is another key predictor for BFV disease. This is consistent with the findings that conditions for BFV outbreaks are dependent on socio-economic status [10]. Tidal inundation of salt marshes is a major source of water for breeding of the coastal mosquitoes. Adult mosquitoes lay their eggs on soil, moist mud, and the bases of plants around the margins of their breeding sites. Our results support findings from previous studies on tidal influence and mosquito distribution [4], [10], [11], [19].

The predictions performed with current data (i.e., 2000–2008) showed that the climatic and socio-economic and tidal variables had the good ability (90·2% accuracy) to predict the probability of future BFV disease risk (Table 1). For prioritising the areas of risk for public health preparedness and policy decision, we examined the future probabilities of risk of BFV disease to differentiate areas of lower risks (0–0·25 probability) from those of higher risks (0·5–1·0 probability) for BFV outbreaks (Figs: 1, 2, 3). Figure 1 indicates that the disease will vary across Queensland in future years. Thus, these results suggest that public health managers and decision makers should increase their surveillance, vigilance, and preparedness in order to control and prevent BFV disease outbreaks in Queensland. In addition, these findings indicate that methods designed to project BFV disease risks in Queensland may be applicable to other regions of Australia.

The projected average increase in temperature in Queensland is likely to be generally favourable for mosquito development and survival, resulting in potentially greater numbers of BFV mosquitoes which in turn may increase BFV disease. However, mosquito survival under these conditions will depend on whether the increased temperatures are accompanied by adequate rainfall. Projected data on rainfall showed variations in annual patterns, particularly in coastal Queensland for the years 2025, 2050 and 2100 [17], thus potentially offsetting the survival benefits provided by increased temperatures in 2025, 2050 and 2100 [17], respectively.

In general, the projections for Queensland show that annual average minimum temperatures are predicted to rise, whereas annual rainfall is predicted to decline [17]. In response to predicted climate change and based on the results of the forecasting model presented here (Fig. 1), there are three possible outcomes of BFV risk for any localised area. Firstly, a reduction in the annual average rainfall may not be sufficient enough to offset the survival benefit of increased minimum temperatures thus resulting in an increase in BFV mosquito and consequently increase in BFV outbreaks. Secondly, a reduction in annual average rainfall exactly offsets survival benefits from increased minimum temperatures resulting in no net gain or loss of BFV mosquito and consequently no change in the frequency or intensity of BFV outbreaks. Thirdly, annual average rainfall reduction is significant enough to reduce the populations of BFV mosquito populations regardless of increasing minimum temperatures thus resulting in a reduction in BFV mosquito populations and BFV outbreaks. In this study, the projections have been based on annual average climatic conditions and the logistic regression model which determined that, on average, projected BFV risk will vary across Queensland compared to existing conditions. However, it is worthwhile noting that the BFV transmission could still be active even if no outbreaks exist.

There are three key strengths in this study. Firstly, to our knowledge, this is the first study to forecast future risk of BFV disease transmission using climate change scenarios. Our analysis provides an insight into future geographic distribution of BFV risk in different areas across coastal Queensland. These scenario-based forecasts can help determine when and where public health interventions are most needed for BFV disease control and prevention. Secondly, an ability to predict interaction between climatic factors of BFV in a given location such as coastal Queensland which is at high-risk is critical for successful future predictions of the risk of BFV outbreaks. Our approach provides a clear link between climatic, socio-economic and tidal factors, and BFV transmission dynamics. Finally, the model we developed had a high level of accuracy, sensitivity and specificity.

This study also has three key limitations. Firstly, in this study, we used only A1B scenario to forecast BFV outbreaks and did not use other scenarios because the goal of this exercise is to identify regions with either reduction or increase in probability of risk of future BFV outbreaks. However, use of different scenarios may be a better approach to understand the range of possibilities (including the worst and best case scenarios) and our future study will evaluate different scenarios. Secondly, we projected risk of BFV only for the coastal regions because of several reasons: 1) previous studies have identified several hotspots of BFV disease along coastal geographic regions [25], [26]; 2) surveillance data indicated that BFV notifications were comparatively higher along coastal geographic regions than inlands [25]; and 3) most of the coastal geographic regions were densely populated (80% of the Queensland population lives along coastal regions) [27]. However, our future study is aimed at examining the outbreak differences between urban and rural areas. Finally, we did not include relative humidity and mosquito density in models because these data are unavailable for this study. Previous studies demonstrated that temperature, rainfall and tides were the most significant risk factors for BFV disease (7, 8). Therefore, we have included these variables in our models. In addition, we did not predict the future outbreaks based on SEIFA as data on projections on the SEIFA index are unavailable at the time of the study.

The potential future risk maps of BFV disease for 2025, 2050 and 2100 revealed the locations of BFV risk with sufficient operational accuracy. In practical terms, the BFV risk map may assist in selecting targeted surveillance sites and guiding preventive control measures. Targeted surveillance and control efforts based on future BFV risk maps should lead to more effective public health interventions prior to the occurrence of outbreaks.

## Conclusions

This study demonstrated the feasibility of developing forecast models using epidemiological, climatic, socio-economic and tidal data for projections of future BFV outbreak risk. These models may have important implications in policy planning and development towards minimising the impact of climate change on BFV disease and other mosquito-borne diseases.

## References

[1] McMichael AJ, Woodruff RE, Kenneth HM, Pizer HF (2008) Climate change and infectious diseases. The Social Ecology of Infectious Diseases. San Diego: Academic Press. pp. 378–407.

[2] Russell RC (2009) Mosquito-borne disease and climate change in Australia: time for a reality check. Aust J Entomol 48: 1–7.

[3] Australian Department of Health and Ageing (2012) Communicable Diseases Surveillance - Highlights. Avaialble: www.health.gov.au. Accessed January 24, 2012.

[4] Dale PER, Breitfuss MJ (2009) Ecology and management of mosquitoes. In: Saintilan N, editor. Australian Saltmarsh Ecology: CSIRO Press. pp. 167–179.

[5] Young J (2009) Strategic directions for communicable disease prevention and control 2009–2012. Brisbane: Queensland Health, Division of the Chief Health Officer. pp. 9–16.

[6] WHO (2004) Using Climate to Predict Infectious Disease Outbreaks: A Review. Geneva: WHO. pp. 55.

[7] I.P.C.C. (2001) Climate change 2001: Impacts, adaptations and vulnerability. Contribution of working group II to the third assessment report of the intergovernmental panel on climate change. Cambridge: Cambridge University Press.

[8] Rogers D, Randolph S (2000) The global spread of malaria in a future, warmer world. Science 289: 1763–1766. 10976072 10.1126/science.289.5485.1763

[9] Hales S, Wet ND, Maindonald J, Woodward A (2002) Potential effect of population and climate changes on global dsitribution of dengue fever: an empirical model. Tha Lancet 1–6. 10.1016/S0140-6736(02)09964-6 12243917

[10] Naish S, Hu W, Nicholls N, Mackenzie JS, Dale P, et al (2009) Socio-environmental predictors of Barmah forest virus transmission in coastal areas, Queensland, Australia. Tro Med Int Health 14: 247–256. 10.1111/j.1365-3156.2008.02217.x 19187524

[11] Naish S, Hu W, Nicholls N, Mackenzie JS, McMichael AJ, et al (2006) Weather variability, tides, and Barmah Forest virus disease in the Gladstone region, Australia. Environ Health Perspect 114: 678–683. 16675420 10.1289/ehp.8568 PMC1459919

[12] I.P.C.C. (2011) Intergovernmental Panel on Climate Change - Publications and Data. Avaialble: www.IPCC-Secwmo.int. Accessed December 7, 2011.

[13] Australian Bureau of Statistics (2011) Census of Population and Housing - Basic community profiles, Commonwealth of Australia, Canberra, ABS.

[14] Australian Bureau of Meteorology (2011) Data obtained from National climate centre, Queensland, Commonwealth of Australia. Avaialble: www.bom.gov.au/climate/. Accessed September 20, 2011.

[15] Queensland Department of Transport and Main Roads (2009) Maritime Safety Queensland:Tides. Queensland official tidal tables and boating safety guide: tidal notes and definitions. Avaialble: www.msq.qld.gov.au. Accessed September 24, 2009.

[16] Australian Bureau of Statistics (2008) Mesh Blocks digital boundaries, Australia. Avaialble: www.abs.com.au. Accessed August 29, 2010.

[17] CSIRO (2011) OzClim Climate change scenario. Avaialble: www.csiro.au. Accessed December 7, 2011.

[18] I.P.C.C. (2011) Contribution of Working Groups I, II and III to the Fourth Assessment Report of the Intergovernmental Panel on Climate Change. Avaialble: www.ipcc.ch. Accessed March 26, 2011.

[19] Jacups SP, Whelan PI, Harley D (2011) Arbovirus models to provide practical management tools for mosquito control and disease prevention in the Northern Territory, Australia. J Med Entomol 48: 453–460. 21485389 10.1603/me10193

[20] Burnham KP, Anderson DR (2002) Model selection and multi-model inference: a practical-theoretical approach. London: Springer-Verlag.

[21] Statistical Package for Social Sciences (2010) SPSS 18.0 Guide for data analysis. New Jersey: Prentice-Hall.

[22] Zhu W, Zeng N, Wang N (2010) Sensitivity, specificity, associated confidencve interal and ROC analysis with practical SAS implementations; Baltimore, Maryland.

[23] Naish S, Mengersen K, Hu W, Tong S (2011) Emerging methods in using GIS to analyse Barmah Forest virus disease in Queensland, Australia. In: Nitin T, editor; New Delhi, India. Teri University.

[24] Lloyd CD (2010) Spatial data analysis. New York: Oxford University Press.

[25] Naish S, Hu W, Mengersen K, Tong S (2011) Spatial and temporal clusters of Barmah Forest virus disease in Queensland, Australia. Trop Med Int Health 16: 884–893. 21481107 10.1111/j.1365-3156.2011.02775.x

[26] Naish S, Hu W, Mengersen K, Tong S (2011) Spatio-temporal patterns of Barmah Forest virus disease in Queensland, Australia. PLoS ONE 6: e25688. 22022430 10.1371/journal.pone.0025688 PMC3192738

[27] Australian Bureau of Statistics (2008) Population by age and sex, Australian states and territories, Jun 2002 to Jun 2007.Cat. no. 3101.0. Avaialble: www.abs.gov.au/AUSSTATS/absnsf. Accessed October 20, 2010.

